# Automated recognition of postures and drinking behaviour for the detection of compromised health in pigs

**DOI:** 10.1038/s41598-020-70688-6

**Published:** 2020-08-12

**Authors:** Ali Alameer, Ilias Kyriazakis, Jaume Bacardit

**Affiliations:** 1 School of Natural and Environmental Sciences, Newcastle University, Newcastle Upon Tyne, NE1 7RU UK; 2 School of Computing, Newcastle University, Newcastle Upon Tyne, NE4 5TG UK; 3 Institute for Global Food Security, Queen’s University, Belfast, BT9 5DL UK

**Keywords:** Engineering, Computational science, Animal behaviour

## Abstract

Changes in pig behaviours are a useful aid in detecting early signs of compromised health and welfare. In commercial settings, automatic detection of pig behaviours through visual imaging remains a challenge due to farm demanding conditions, e.g., occlusion of one pig from another. Here, two deep learning-based detector methods were developed to identify pig postures and drinking behaviours of group-housed pigs. We first tested the system ability to detect changes in these measures at group-level during routine management. We then demonstrated the ability of our automated methods to identify behaviours of individual animals with a mean average precision of $0.989 \pm 0.009$, under a variety of settings. When the pig feeding regime was disrupted, we automatically detected the expected deviations from the daily feeding routine in standing, lateral lying and drinking behaviours. These experiments demonstrate that the method is capable of robustly and accurately monitoring individual pig behaviours under commercial conditions, without the need for additional sensors or individual pig identification, hence providing a scalable technology to improve the health and well-being of farm animals. The method has the potential to transform how livestock are monitored and address issues in livestock farming, such as targeted treatment of individuals with medication.

## Introduction

There are increased concerns by consumers and the livestock industries themselves over the sustainability of livestock systems. These concerns include the well-being of the animals and the use of medication^1^. Health compromises in commercial pigs are a significant concern for both welfare and productivity, and are associated with significant use of antimicrobials^2^. Disease, both clinical and subclinical, is the main factor responsible for decreases in performance and a challenge to the sustainability of pig systems^3^. Early detection of health and welfare issues is required to allow timely intervention, mitigate losses and improve well-being^4^. On a commercial scale, the observation of subtle changes by humans in behaviour that may accompany subclinical or early-stage clinical disease is impractical^5^. Automated detection of these changes through cheap and scalable technologies, therefore, is required. To focus efforts, identifying the appropriate behaviours to monitor—from which we can extract the most valuable information—is imperative, taking into account commercial stocking density and standard pen layouts. Changes in posture^6^ and drinking behaviour^7^ are key indicators of health compromises and reduced welfare, and development of automated systems, which are effective at monitoring these behaviours, are likely to be of greatest value.

Video surveillance is a suitable technology to identify drinking^8^ and feeding^9^ behaviour in pigs, due to its low cost and the simplicity of its implementation, and a good alternative to Radio-frequency identification systems installed around drinking systems and feeding stations^10^. The crucial element of this approach is how to extract formative features from the images^11–13^, i.e. that can provide appropriate information regarding the health of the pigs in a manner that has relevance to the pig keeper. In recent years there have been relevant attempts on how to obtain accurately animal behaviours with various machine learning methods^14–17^. Depth sensors have been utilised to accurately track pigs and identify their standing and non-standing behaviour^10,18,19^. However, these approaches have not been capable of identifying specific postures of relevance, within the non-standing groups, e.g., sitting, lateral and sternal lying.

Inexpensive RGB (red, green and blue) cameras have been used to distinguish the pigs from their background using handcrafted filters of feature extraction, e.g., Gabor filters^20^. The main drawback for these image processing methods is their inability to cope with the variable farm environment (e.g., varied illumination) that may easily disrupt system performance. To tackle these challenges, convolutional neural networks (CNNs) have been proposed to accurately detect pigs^21–25^. The dynamic filter selection in CNNs enables these methods to achieve invariance to different farm conditions^26,27^. A limitation associated with existing deep learning based methods includes the requirement for pigs to be individually marked, adding unscalable labour burden to mark the pigs. Additionally, spray marks can become less visible and repeat marking may be required. Another challenge associated with the above approaches, is their dependency to track pigs in order to estimate behaviours. The challenging farm environment can frequently disrupt system performance, e.g., when a pig moves beyond the camera field of view.

Here, we propose a whole-encompassing system that identifies not only basic behaviours, such as standing and non-standing, but also more complex postures, e.g., sitting, lateral lying, sternal lying and drinking behaviour, and mean speed and distance travelled using inexpensive RGB cameras. Several of these behaviours may have diagnostic value in pigs: for example, sternal or ‘belly lying’ may be indicative of a pig being cold or suffering from abdominal pain^5^. Dog-like siting behaviour may be indicative of locomotory problems^2^. Changes in the frequency and duration of drinking behaviour, are frequently an early indicator of infection^5^. We carried out a controlled study, monitoring the behaviour of growing pigs in a commercial environment. Their standard husbandry routine was disrupted, by giving them restricted access to feed at pre-defined time periods to change their normal behavioural pattern in a time-controlled manner. This created a scenario that was akin to a fault with an automated feeder. The behavioural data collected during these periods of routine and of disruption was used to develop our methodology for automated recognition of key postural and drinking behaviours. To the best of our knowledge, no previous attempt has been made to detect said high-level behaviours (e.g., sitting) of commercial pigs (at both group-and individual-level) based on 2D cameras.

## Results

### Group-wise measures for the food restriction experiment

**Figure 1:**
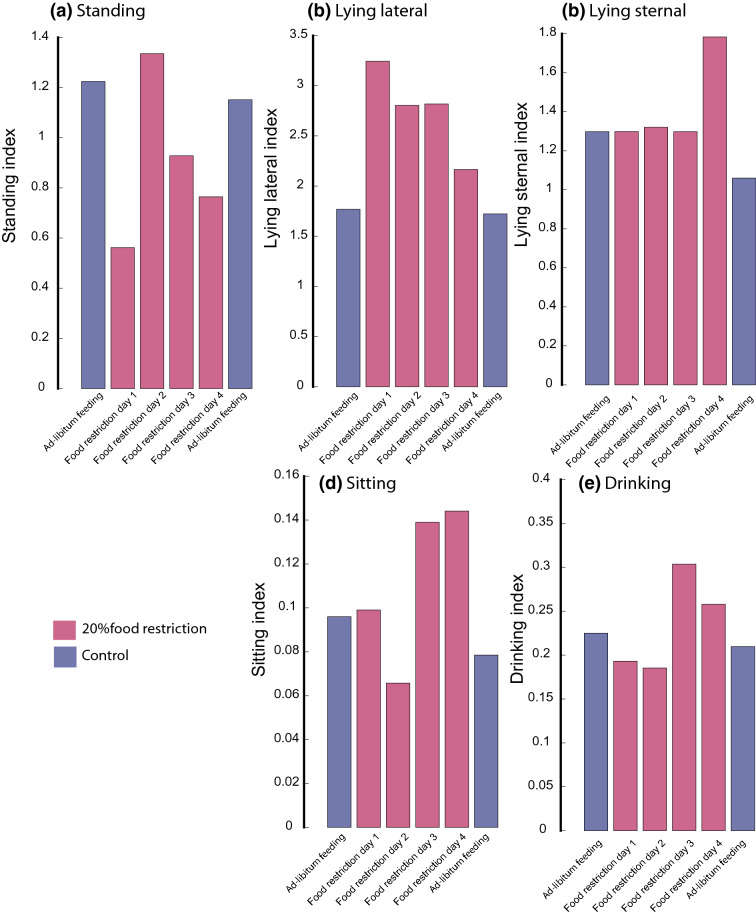
The scored (**a**) standing, (**b**) lateral lying, (**c**) sternal lying, (**d**) sitting and (**e**) drinking indices per day (11:00–15:00) across the study period. The indices were calculated to provide consistent measures across various data frames. Control bars represent indices averaged across 2 days immediately before and after the food restriction period. These metrics were obtained using the developed/validated primary model.

By restricting access to food at designated time points, changes in both drinking behaviour and posture were observed. Such changes were expected to be beyond the extent of behaviours observed under ‘undisturbed’ conditions. Indeed, food restriction decreased the amount of standing, and increased the amount of lateral lying and sitting. Initially, when food was restricted, a 53.3% decrease in the standing index was observed compared to the preceding days, when food was available *ad-libitum* (baseline). During the second test day, the standing index returned to levels similar to baseline, but then again decreased during days 3 and 4 of food restriction relative to baseline ($-\,23.3$% and $-\,36.6$% respectively) (Fig. 1a). Conversely, the lateral lying index showed a 45.3% increase compared to baseline on day 1 of food restriction. During the subsequent 3 days, the lateral lying index decreased somehow, but remained above baseline levels for the entire test period ($+\,35$% over baseline on day 2 and 3, $+\,20$% on day 4) (Fig. 1b).

Across the first 3 days of food restriction, no change was observed in the sternal lying index compared to baseline. However, a substantial increase (36.9%) was observed in the sternal lying index on the 4th day of restriction compared to baseline. The sitting index showed the most substantial change from baseline on days 2–4 of food restriction, with a decrease of 33.2% on day 2 and an increase of $+\,35$% compared to baseline on days 3 and 4. The drinking index showed substantial change on day 3 of food restriction with a 31.7% increase (Fig. 1c–e).

Following the training and testing phases, our proposed methods, namely, you-only-look-once (YOLO)^28^ and Faster Regions with CNN features (Faster R-CNN)^29^ combined with the deep residual network (ResNet-50), identified pigs (pen wise; Fig. 1) that exhibited drinking behaviour and postures, i.e., standing, sitting, lateral lying and sternal lying across the study period.

### Estimating anchor boxes

**Figure 2:**
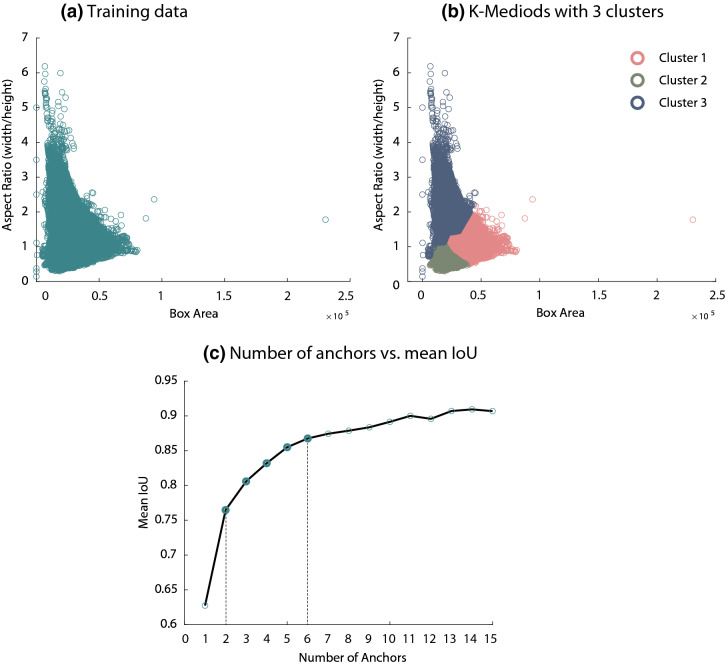
Estimation of anchor boxes using the K-Medoids clustering algorithm. (**a**) shows the ground truth box distribution of our training data with respect to box aspect ratio and box area. (**b**) visualises three clusters, obtained using the K-Medoid algorithm, from which three anchor boxes were selected. (**c**) exhibits the trade-off between the number of anchor boxes (a maximum of 15; x-axis) and the mean *IoU* (y-axis) of each subset anchor boxes.

The visualisation of the ground truth box distribution of our training data (with respect to box aspect ratio and box area) has revealed a wide variation across the aspect ratio (Fig. 2a). Using the proposed mechanism of clustering, led to boxes of similar areas and aspect ratios to be grouped proportionally, (Fig. 2b). As shown in (Fig. 2c), increasing the number of anchors lead to an improved mean intersection-over-union (*IoU*) measure, hence it better represents the training dataset. It can also be noticed, in (Fig. 2c), that there is only a slight improvement in mean *IoU* beyond six anchor boxes. Given these results, we evaluated both of our detectors using values between 2 and 6 (see Supplementary Table S1), to finally determine the number of anchor boxes required to satisfy the required trade-off between detection speed and accuracy.

### Performance of the pig identification and tracking models

**Figure 3:**
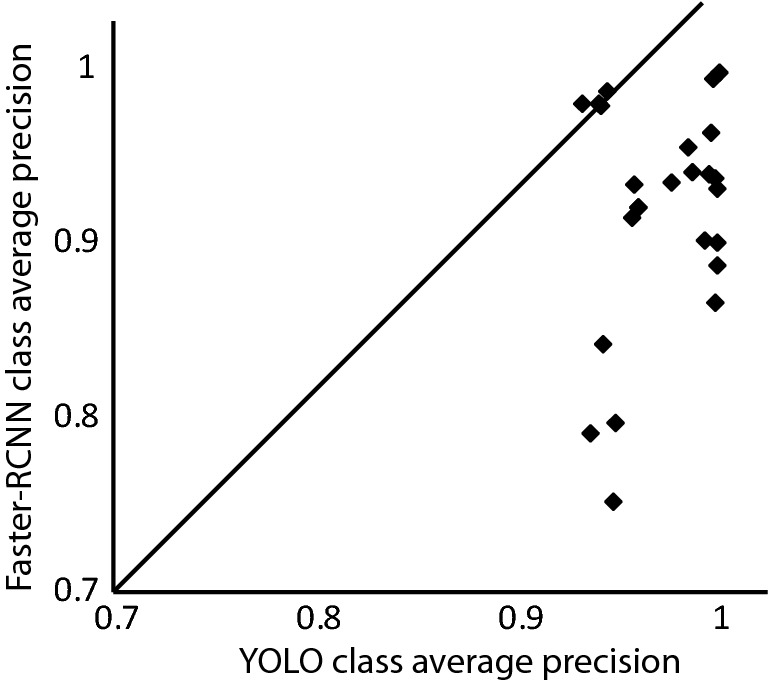
A comparison between the average class precision of YOLO and faster R-CNN detectors. Markers of the scattered diagram represent the classes of both detectors with a specific anchor box. Classes below the diagonal indicate that the YOLO detector outperforms the faster R-CNN detector.

Table 1 displays the results for trained detectors across the selected range of anchor boxes. The table also provides the mean average precision and the detection (inference) speed for each scenario. To select a primary model to quantify pig behaviours during our study period, we compared the per-class average precision of both utilised detectors across proposed anchor boxes (Fig. 3). The majority of classes detected with YOLO have had higher average precision

**Table 1:** The mean average precision (mAP) ± standard deviation (SD) and the detection (inference) speed/image ± standard deviation (SD) for YOLO and Faster R-CNN detectors across all proposed anchor boxes. The mean speed was calculated/averaged across 1,000 selected images.

Anchor boxes	YOLO		Faster R-CNN	
	Mean average precision ± SD	Speed per image ± SD	Mean average precision ± SD	Speed per image ± SD
2	0.9695 ± 0.0269	$0.0112 \pm 2.1 \, \hbox {x} \, 10^{-4}$	0.8987 ± 0.0713	$0.0419 \pm 1.2 \, \hbox {x} \, 10^{-4}$
3	**0.9888** ± **0.0094**	$\mathbf{0.0128} \pm \mathbf{1.5} \, \hbox {x} \, {\mathbf {10}}^{-\mathbf{4} }$	0.9054 ± 0.0580	$0.0432 \pm 6.3 \, \hbox {x} \, 10^{-4}$
4	0.9674 ± 0.0282	$0.0131 \pm 2.8 \, \hbox {x} \, 10^{-4}$	**0.9149** ± **0.0524**	$\mathbf{0.0440} \pm \mathbf{2.9} \, \hbox {x} \, {\mathbf {10}}^{-\mathbf{4} }$
5	0.9663 ± 0.0288	$0.0144 \pm 3.7 \, \hbox {x} \, 10^{-4}$	0.8919 ± 0.0842	$0.0445 \pm 7.5 \, \hbox {x} \, 10^{-4}$
6	0.9657 ± 0.0295	$0.0158 \pm 3.3 \, \hbox {x} \, 10^{-4}$	0.9039 ± 0.0766	$0.0476 \pm 8.6 \, \hbox {x} \, 10^{-4}$

.

Due to the high performance, i.e., mean average precision ($mAP = 0.98$) and speed (0.012 *s*/*image*), of the YOLO model trained using three anchor boxes, this model was selected as the primary platform for extracting animal behaviours during the food restriction protocol period. Table 2 shows that this detector achieved high average precision and low average miss-rate across the studied pig behaviours of different trials. When tested in different experimental conditions (Table 2), the primary model showed consistent performance with a high mAP (0.9698).

**Table 2:** The average class precision and average class miss-rate for our primary model, YOLO detector with 3 anchor boxes. This model achieves high precision and low miss-rate across classes of both: the food-restriction trial and an independent commercial pig trial. The drinking sources were outside the camera field-of-view in the latter trial.

Class	Food-restriction trial		Independent commercial trial	
	Average precision	Average miss-rate	Average precision	Average miss-rate
Standing	0.9845	0.0196	0.9867	0.0204
Sitting	0.9864	0.0130	0.9012	0.0982
Sternal lying	0.9968	0.0049	0.9929	0.0070
Lateral lying	0.9998	0.0004	0.9984	0.0017
Drinking	0.9766	0.0093	–	–

Further to its high detection performance, the primary model exhibited high multi-object tracking accuracy (*MOTA*) and precision (*MOTP*) of 0.94 and 0.80, respectively, with a true positive rate of $99.97\%$. Compared with the ground truth tracking dataset, it achieved a low error-rate in obtaining the total distance travelled and average speed. We used the mean squared error (*MSE*) to quantify the performance of the locomotor activities, i.e., (0.078 *MSE*, total distance travelled; 0.002, average speed).

**Figure 4:**
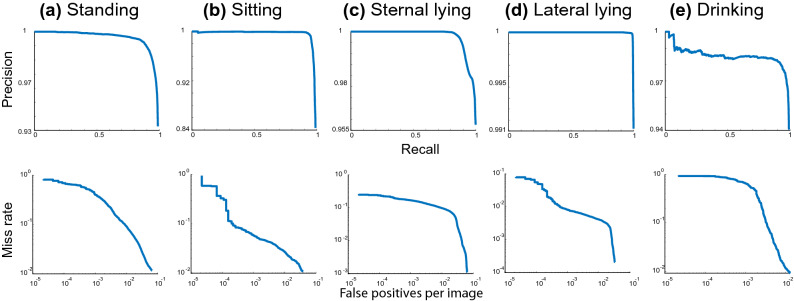
The performance of our primary model. Upper row: precision-recall curve. A point on the precision-recall curve is determined by considering all pig behaviours detected with a given threshold as positive predictions, then calculating the resulting precision and recall for that threshold. Below row: miss rate against FPPI. This characteristic curve obtained with similar mechanisms of the PR curve however to represent miss detected pigs; both axes are logarithmically scaled.

The precision/recall (PR) curve shows that our primary model achieved high precision at varying levels of recall, see (Fig. 4; first row) and (Supplementary Figure S1; first column). The PR curve also indicated that for all animal behaviours, our primary model did not compromise the false positive rate, i.e., increasing the number of detected pigs (behaviours), to maintain a high recall, i.e., detecting all ground-truth pigs (behaviours). Similarly, we plotted the miss-rate changes against the number of false-positives per image (*FPPI*) by varying the threshold on the detection confidence, see (Fig. 4; second row) and (Supplementary Figure S1; second column). The curve shows that all classes (e.g., lateral lying) generated low miss rates at lower values *FPPI*, e.g., $10^{-2}$.

**Figure 5:**
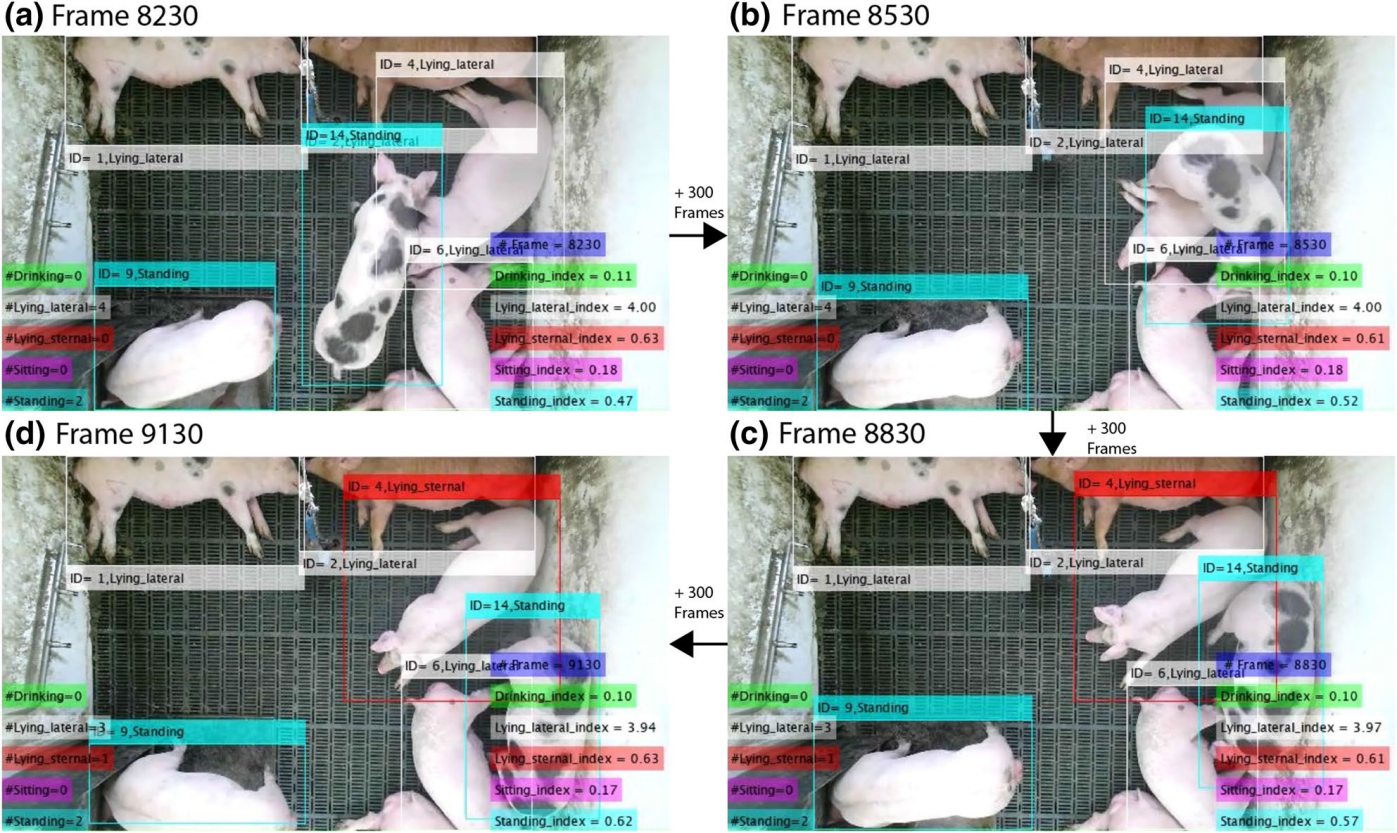
Example of detecting pig postures across a period of 900 consecutive frames, with an interval of 300, using our primary model. The proposed model generated indices to each pig behaviour; it also assigned a vID to each pig to associate these across frames. The images in the figure were extracted from our dataset (‘Methods’ section).

Figure 5 illustrates an example of detecting postures across a period of 900 consecutive frames. For each frame, we recorded the number of pigs exhibiting one (or more) of the five targeted behaviours. Then, we instantly associated an index as a function of the corresponding cumulative behaviour and the total number of frames, see Eq. (1). This mechanism ensured that we have consistent measures across various data frames, e.g., recording drops frames due to hardware overheating issues.

We also allocated a virtual unique identification (vID) to each pig in each frame. This not only allowed tracking individual pigs in the two-dimensional space, but also recorded their associated behaviours across time, e.g., lying locations^30^. For instance, the pig with ID=4 changed its posture from lateral lying at frames (8,320, Fig. 5a; 8,530, Fig. 5b) to sternal lying (8,830, Fig. 5c; 9,130, Fig. 5d). During this transition, we ensured that the pig maintained its virtual identity.

## Discussion

We developed a system capable of automatically detecting changes in key posture and drinking behaviours exhibited by pigs in a commercial environment. These changes may be subtle indicators of reduced health and welfare that cannot be simply observed at pen side by farm staff^2^. Our system provides high behaviour detection precision and speed. This can confer sustainability benefits to farmers through enhanced production, improved animal welfare and timely action, i.e. use of medication. Overall, our work makes several contributions to the automated detection of behaviours of commercially housed pigs:

1. We developed a whole-encompassing system that detects high-level pig postures, such as sitting and drinking behaviour, using RGB cameras.
2. To improve upon the speed and precision of existing pig detection and behaviour estimation methods^31^, we reframed the goal of the method to directly obtain pig behaviours from images. This was instead of first detecting pigs and then inferring the corresponding behaviour at different stages^21,32^.
3. We investigated the characteristics of two well-known detectors^28,29^, in terms of speed, pig behaviour detection precision and miss rate, showing that YOLO is clearly superior for this task. We implemented a data-driven process to identify the optimal layer of our backbone network (ResNet50) for feature extraction. Then, we assessed the relevance of different sets of anchor boxes (obtained with the K-medoid clustering algorithm).
4. In addition to detecting group level drinking and postures, we generated individual profiles for pigs, which included movement history and the corresponding behaviour at each point in the time-space.
5. We showed that the proposed method can detect group-level behavioural changes following a disruption to the pig routine (feeding regime).
6. Produced and made publicly available a large annotated dataset for pig identification, tracking and behaviour detection.

In the following subsections we discuss the contributions and results of this work from a variety of perspectives.

**Figure 6:**
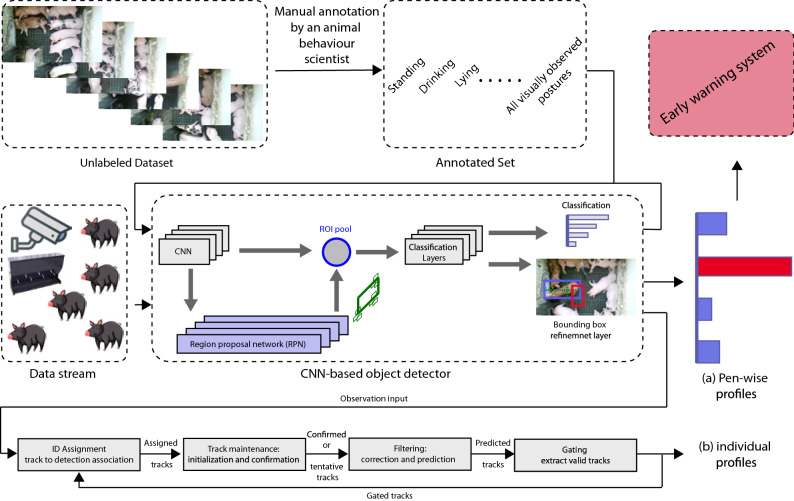
Architecture proposed for the automatic recognition of postures and drinking behaviour in commercially housed pigs. Each pig behaviour within the selected frames of our dataset was annotated by an animal behaviour scientist. The architecture above is capable of generating (**a**) group-level profiles for each pen; (**b**) individual profiles for each pig that include their postures, drinking and locomotion activities. Only pen-wise profiles were used to detect changes in behaviour at group-level during the feed-restrictions protocol period. The images in the figure were extracted from our dataset (‘Methods’ section).

### Behavioural observations

When food was initially restricted, a decrease in standing and an increase in lying indices were observed, which was likely to be linked to the reduced presence of resources (feed) for the pigs to access and the animals having to wait for a cue that food is again available, i.e. the arrival of a stockperson. In the initial stages of feed restriction when there is limited impact from hunger, the pigs appeared to wait for such cues before performing any anticipatory-type behaviours^33^. However, over the 4-day period, the standing index increased, as pigs were becoming hungrier and were likely searching for additional resources. The sitting index showed a sharp increase on restriction days 3 and 4, where pigs were more active and could be demonstrating anticipatory behaviour^33^. Following the initial decrease in drinking when food was first limited, a subsequent increase in drinking was observed. This could be the outcome of compensatory consummatory behaviour, where pigs drink more in the absence of the required amount of food^34^. All these changes highlight key behaviours which may be of value for identification of impaired health and welfare^2,5^.

**Figure 7:**
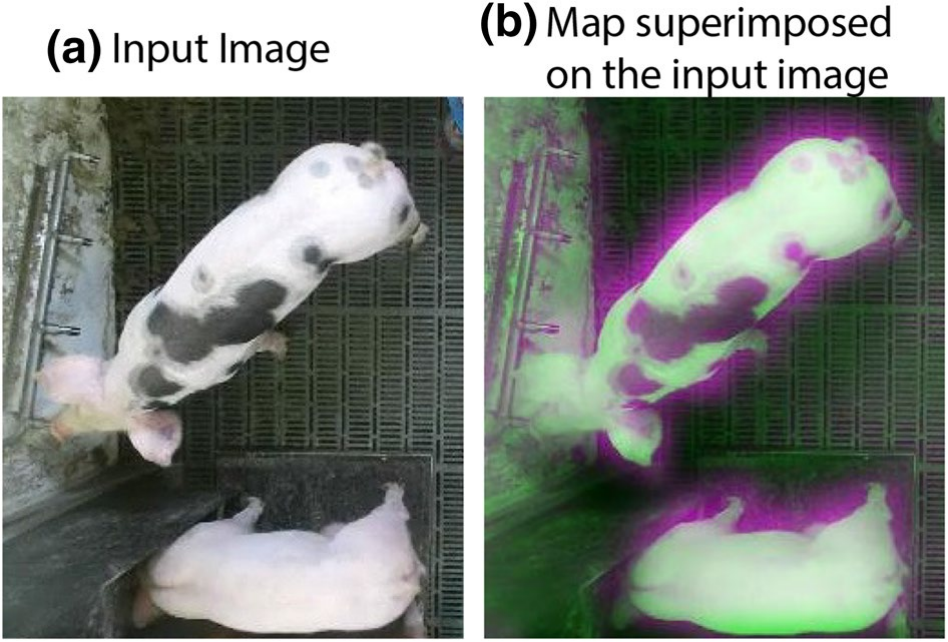
An example of (**a**) an input image with (**b**) the corresponding activations of the base model of the primary platform, i.e., YOLO detector with three anchor boxes. This visualisation technique highlights the areas of the image that drive the method to detect pig behaviours, standing in this specific example, showing how this model makes decisions and (potentially) identifying confounders. The images in the figure were extracted from our dataset (‘Methods’ section).

In this work, we selected an inclusive set of pig behaviours whereby behavioural scientists routinely quantify on a frame-by-frame basis. We did not quantify pig feeding behaviour, as the feeders were outside the camera field of view. However, we recognise that changes in feeding behaviour are one of the most common indicators of compromised health and welfare^5,35^. In order to extrapolate this approach to encompass feeding associated behaviours, we may define new classes of images, such as the feeding and non-nutritive visit (foraging) behaviours^35^. Practically, this can be done by either utilising transfer learning (i.e., storing knowledge gained while adding extra-classes), or by redefining the dataset and following the methods described in this paper.

### Individual pig profiles

**Figure 8:**
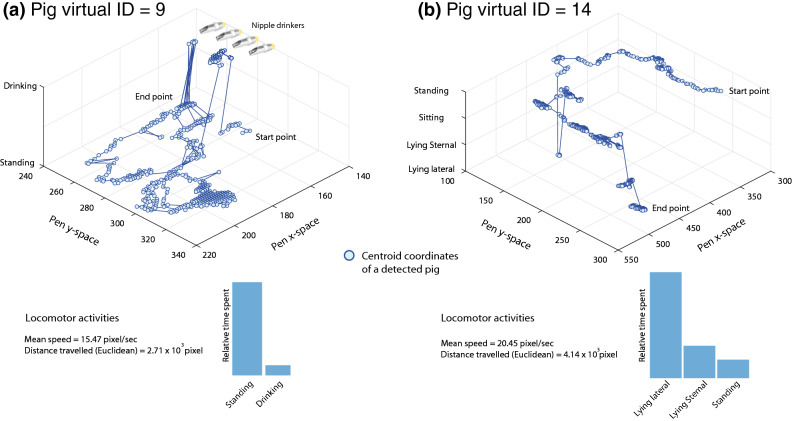
Three-dimensional trajectory of individual pigs: (**a**) vID = 9, (**b**) vID = 14. Each point represents the centroid of the detected pig in the pen x,y space. The z-axis expresses behaviours at each point in time, e.g., drinking, standing, sitting, sternal lying and lateral lying. Bar chart represents the relative proportion of time a pig exhibited a given behaviour.

For each pig in a given frame, we allocated a unique vID that includes pig locations and exhibited behaviours. To depict a pig position, we used the bounding box centroid. Processing individual profiles of pigs enabled quantifying the relative time spent performing postures and/or drinking behaviour; it also enabled obtaining locomotor activities, e.g., mean speed and distance travelled of individual pigs. The example in (Fig. 8a) shows that pig with vID = 9, spent a relatively large proportion of time standing. This pig also approached the drinking source on a few occasions and consequently exhibited drinking behaviour. With this projection, we may spatially validate pig behaviours, e.g., the drinking behaviour at the vicinity of drinking source. Another interesting observation is that the location of pigs varies only when the detected behaviour is standing. Similarly, in (Fig. 8b), the pig with vID = 14, spent a large proportion of time lying laterally. Pigs identified as lying were shown to be stationary across time; this was demonstrated in (Fig. 8) with a large number of observations clustering in a small approximation. The above observations may endorse the system precision intuitively.

The ability to detect individual animals is beneficial; however tracking multiple animals in close proximity at commercial stocking densities is challenging due to, e.g., the similar appearance of different pigs^36^. To tackle these problems, we developed the method to score pen-wise behaviours on a frame-by-frame basis. Generating behaviours on this basis enabled maintaining accurate detection even when the frame-sequence is disrupted, sensors dropping frames due to , e.g., overheating or congestion in the data capture infrastructure. This overcomes problems with existing methods^31^, whereby each behavioural detection requires a large number of consecutive frames with a high frame rate. Generating behaviours on this basis also prevents short-term tracking segments, e.g., $<\,20$ min^18^ (due to a pig leaving the camera filed-of-view and occlusion), from compromising system performance. To generate individual profiles, a separate processing stage was designed to perform pig tracking (Fig. 6b). Our method of measuring standing and lying postures is data-driven and does not require calibration, such as setting thresholds of depth^10^ or the number of segments^37^. The method learns the task only by using many examples of pig images exhibiting said behaviours. The proposed system does not put particular emphasis on a pig’s head to identify behaviours; the obscuration, i.e., self-occlusion, to pig heads would prevent sustainable detection of several behaviours. Instead, our method detects pig behaviours based on the whole structural features of the pig even when the head is entirely invisible. This is an advancement over previous work addressing the same challenge^21,23^.

### Pig detection and visualisation

From a technological perspective, the selection of anchor boxes has a great impact on system performance as the obtained shapes/sizes adapt better to the dataset, e.g., size of the pigs^38^. Here, we used a clustering method, K-medoids with the intersection-over-union as a distance metric, to estimate the dimensions of boxes that effectively represented our dataset. The K-medoid generated more representative boxes that are less sensitive to outlier bounding boxes, due to using the medoid of clusters rather than e.g., the mean. Similarly, the use of *IoU* distance metric provided more accuracy in generalising to pigs with different sizes; other metrics, e.g., Euclidean distance, produced a larger error when they encountered larger box sizes. This is demonstrated in (Fig. 2b), where boxes of similar aspect ratio and sizes were clustered proportionally. The YOLO detector showed superior performance when compared to Faster R-CNN detector. Detecting relatively large objects, such as pigs, is deemed more compatible with YOLO detector where it learned generalisable representations of pigs within its context by extracting features from the entire image^28^. Finally, we inspected specific inner parts of the ResNet50 base model that activate when identifying pig postures an on what parts of the source data they rely on. In (Fig. 7), we show an example of an input image (Fig. 7a) superimposed by the baseline-network activation (Fig. 7b). This visualisation shows that the model relies on informative features that correspond to pig postures and drinking behaviour, e.g., legs, contours and drinking sources.

## Concluding remarks

Changes in postures and drinking behaviours are key indicators of health compromises and reduced welfare in pigs^5,10^; accurately quantifying these are of great value for early intervention^39,40^. We demonstrated that such changes can be detected automatically through the methods developed in this paper. Using a controlled trial where pigs were food restricted for a 4-day period, we induced subtle changes in specific postures and drinking that can be used for detection of food restriction, e.g., due to equipment malfunction. Such changes are impossible to detect at pen side by a stockperson, which warranted the development of this system that, as our experiments show, can monitor and detect such important changes in the patterns of postures and drinking behaviour.

Additionally, we demonstrated that our system is able to generalise to tackle challenging conditions, e.g., fluctuations in natural lighting and pig size, which are common in working farm environments. In computer vision, validating with larger data is essential, as smaller datasets may overfit and therefore produce over-optimistic results^27^. To date, ours is the largest labelled dataset used in the field of precision pig farming; researchers may utilise it as a workbench in developing methods of pig behaviour detection.

Recent advancements focus on monitoring behaviour where the task of detecting problems correspond to users of the system^4^. Developments towards systems that automatically alert staff to behavioural changes are limited to limited behaviours, e.g., lying (or not)^41^, which maybe arbitrary^21,23,37^. Changes in relevant behaviours are impractical to quantify manually and early detection, through automation, allows for timely intervention to prevent a further reduction in animal welfare and associated economic losses. This paper proposed a novel solution to resolve existing problems in automating the detection of behaviours in pigs. We identified behaviours with diagnostic validity from a reproducible framework comprising a controlled behaviour study and automated behaviour monitoring system that is practical and relevant to commercial livestock. Changes, or combinations of changes, in behaviours obtained in this framework may precede subclinical and clinical signs that have diagnostic value for a whole-encompassing early warning system^4^. Our work also has a broader significance, in relation to the one-health concept where human, animal and environment health are all linked together^42^.

## Methods

All procedures were conducted in accordance with the Animals (Scientific Procedures) Act 1986, European Directive EU 2010/63, with the approval of Animal Welfare and Ethical Review Body (AWERB) of Newcastle University^10,35^.

### Animals and experimental design

Fifteen pigs (Landrace/Large White dams $\times$ synthetic sire line, Hermitage Seaborough Ltd., North Tawton, UK) were housed, under commercial conditions, in a single, fully-slatted pen ($\hbox {4m} \times 2.4 \, \hbox {m}$) from 9 to 14 weeks of age^35^; this pig density is equivalent to that required by UK commercial conditions (MAFF 1994). Ear tags allowed pigs to be individually identified. Food and water were provided to the pens using 4 drinking sources and 4 feeding troughs respectively^10,35^. To match commercial enrichment standards, a hanging chain with plastic pipes was also provided^35^. All pigs had been previously vaccinated against pneumonia at 7 and 28 days of age (1 mL M + PAC each injection, MSD Animal Health, Milton Keyes, UK), post-weaning multi systemic wasting syndrome at 28 days (1 mL CircoFLEX, Boehringer Ingelheim GmbH, Ingelheim, Germany), and Glässer’s disease when 9–10 weeks old (2 mL Porcilis Glässer vaccine, MSD Animal Health., Milton Keyes, UK)^10,35^. During the study period (Summer time), the mean ambient temperature was $26.3^{\circ } \hbox {C}$ (range: $21.9\hbox {-}28.3^{\circ }\hbox {C}$) and the relative humidity varied from 41 to 54% (mean 47%)^35^. Pigs were weighed weekly for the duration of the study, with a mean start weight of 50.0 kg (±1.22 SEM). Pigs were fed *ad-libitum* a commercial food appropriate for their stage of growth. Food that remained in the feeding troughs was removed, weighed and replaced, with a predefined amount of new food at approximately 09:30 of every morning. The *ad-libitum* protocol applied for 4 days. Subsequently, pigs were quantitatively food-restricted with the pen receiving 80% of their daily *ad-libitum* feed for 4 consecutive days at 12 weeks of age. Food allocation at 12 weeks of age consisted of *ad-libitum* daily intake: 0.059–0.070 kg feed/kg initial total pen body weight; restriction daily intake: 0.047 kg feed/kg initial total pen body weight. Immediately following the 4 days of food restriction, pigs were returned to *ad-libitum* feeding. No adverse effects were recorded at any point during the food restriction protocol. After completion of the study, all the pigs were checked by a veterinarian and released back into the commercial stock^10,35^.

### Equipment set-up

We used a bespoke low-cost data acquisition and processing hardware/software solution that was designed to capture a variety of data (not only video footage). The floor area of the pen was captured with RBG cameras (Microsoft Kinect for Xbox One, Microsoft, Redmond, Washington, USA) attached to the ceiling within ingress protected enclosures and positioned perpendicularly to the pen floor^43^. The camera field of view covered a large space of the pen including the drinking sources, but not the feeding troughs. Videos of pig behaviour were recorded at $\sim$ 25 frames per second with image frame width of 640 pixels and frame height of 360 pixels. Due to the long/continuous recording period in a harsh farm environment and limited processing capacity, this infrastructure often dropped frames from the captured footage.

### Dataset and ethogram

Sample frames were selected from our database of video sequences to construct a data set for training and testing. Our behavioural dataset comprised a total of 7,35,094 instances (an instance denotes an individual pig label with its bounding box coordinates in a given image) across 1,13,379 images; each pig within an image was manually annotated into one of five categories (see Table 3). By definition, the bounding box specifies the location of a pig within an image. It contains a vector in the format [x y width height], where x and y correspond to the upper left corner of the bounding box while the width and height denote width and height of the rectangular-shaped box around each pig. The classes of the dataset were: Standing (1,05,132 instances across 54,320 images), Sitting (23,801 instances across 22,417 images), Lateral Lying (4,17,134 instances across 1,05,199 images), Sternal lying (1,66,085 instances across 81,495 images) and Drinking (22,942 instances across 19,208 images).

To quantify behaviours across the study period, we calculated their indices as the following:

1 $$\begin{aligned} BH_i = \frac{\sum _{k=1}^{N}NBF_k}{N} \end{aligned}$$

in Eq. (1), $BH_i$ refers to a given behaviour index, e.g., standing index, *N* is the total number of frames in a video segment, $NBF_k$ is the number of pigs exhibiting behaviour (*BH*) at the $k{\mathrm{^{th}}}$ frame. We utilised said indices to ensure consistent measures across various data frames. Indices were scored between 11:00–15:00, 2-days immediately prior to the period of restriction, the 4 days of food restriction and 2-days immediately following the restriction period. This time period was selected as the pigs were left undisturbed during these hours and all routine welfare checks and husbandry, including feeding, occurred outside these times. The collected image dataset was annotated by an expert animal behaviour scientist and encompassed a variety of scenarios, for example, pigs standing on top of each other and pigs in direct contact with one another with different illumination conditions. The entirety of the dataset was formed using several short-period footages selected from different days/time-of-the-day throughout the trial period. As a result, the dataset used in this work is diverse and representative of a standard commercial pig pen. We configured a set of pre-processing stages to augment our dataset, applying random horizontal flipping and arbitrary scaling. We also altered the colour of the pixels with selected values of saturation, brightness, and contrast using the HSV colour space^44^. We generated this additional augmented dataset to train our system to generalise to extreme scenarios of e.g., high exposure to sun-light. We designed procedures for facilitating the annotation of the data necessary to train the proposed methods. To automate the annotation process between consecutive video frames, we utilised Kanade-Lucas-Tomasi (KLT)^45^, a feature-tracking algorithm, to track a set of points of pigs in a video.

**Table 3:** Definitions of the behaviours recorded in the dataset.

Behaviour	Definition
Standing	Pig has feet (and possibly snout) in contact with the pen floor
Sitting	Only the feet of the front legs and the posterior portion/bottom of the pig body are in contact with the floor
Lateral Lying	The side of the trunk of the pig is in contact with the floor
Sternal Lying	The chest / sternum of the pig is in contact with the floor
Drinking	The pig snout is in contact with a nipple drinker

To validate the performance of the behaviour detection-system against different experimental conditions, we collected and annotated another dataset from a different commercial pig trial that was carried out during a different season (springtime), resulting in changes in natural light between datasets. Variations in the data also include new pen areas, e.g., position and type of feeding troughs, and pig sizes i.e., mean weight of pigs: 34.17 kg. The classes of the dataset were: Standing (10,151 instances across 4,000 images), Sitting (275 instances across 275 images), Lateral Lying (11,028 instances across 4,000 images) and Sternal lying (6,018 instances across 4,000 images).

To test the capabilities of our system to track individual pigs within a commercial environment, we annotated a dataset specifically for tracking. Instead of annotating pig postures in each frame, unique identification numbers (IDs) were given to each pig across frames. This dataset consisted of single video footage with 2,000 frames and 11,952 instances. We generated this dataset to quantify the characteristics of our tracking method in relation to maintaining a consistent trajectory and the alignment between the annotated and the predicted bounding boxes.

### Proposed methodology

We used the pipeline in (Fig. 6) to train and validate our system. The annotated dataset, provided by the animal behaviour scientist, was fed to the developed CNN-based behaviour detector for training. In this work, we adopted two standard architectures of anchor-based object detectors, YOLO^28^ and Faster R-CNN^29^ due to their high detection precision and speed. The above anchor-based object detectors have achieved high mAP and speed on standard detection tasks like PASCAL VOC^46^ and Microsoft COCO^47^. We also proposed strategies for selecting anchor boxes for both methods. For both detectors we used the same environment, e.g., base feature extraction model, anchor boxes and other hyper parameters.

To preserve the identity of pigs across consecutive frame sequence, we used Munkres variant of the Hungarian assignment algorithm^48^. This method enabled us to determine which tracks are missing and which detections should begin new tracks. To estimate the location of missing tracks, we utilised the Kalman filter^49^. Then, we associated the detected labels (e.g., standing) with the assigned pig ID that was subsequently used to generate individual pig profiles.

Models were implemented in Matlab R2019b on core i9 processor (4.3 GHz) PC using ($8 \times 16$) G RAM and NVIDIA GeForce RTX 2080 Ti GPU.

#### Pig detection method

For both Faster R-CNN and YOLO, we used ResNet-50^50^ as a base model. The rationale for selecting the residual network architecture is the reduced prediction time and its relatively small network size. The prediction time for processing a single image is 0.01 sec, and the total size of the network is 96 MB. Furthermore, this network architecture achieves high accuracies and low error rate on well-known datasets in machine vision such as ImageNet dataset^51^. Studies show that this network architecture is also robust when embedded within the architecture of anchor-based detectors, i.e., YOLO^28,38^ and Faster-RCNN^28,29^.The network depth, defined as the largest number of sequential convolutional or fully-connected (FC) layers on a path from the input layer to the output layer, is 50 layers with around 25.6 million parameters. We selected the hyper-parameters (e.g., solver, learning rate schedule settings, batch size, and the maximum number of epochs) for training the network using nested cross-validation. Finally, a softmax layer was utilised to perform the classification predictions. We leveraged a transfer learning strategy by pre-training our base network on the very large ImageNet database^51^.

The $40{\mathrm{^{th}}}$ relu layer of ResNet-50 model was selected for feature extraction based on an empirical analysis with nested cross validation. This layer generates feature maps that are down sampled by a factor of 16. This amount of down sampling provided a good trade-off between the strength of the extracted features and spatial resolution. Features extracted in deeper layers of the network encode higher-level image details, however, at the cost of spatial resolution.

The YOLO object detector uses a single-stage object detection network to make the process faster. In this work, we used the second version of the YOLO model^28^, as it is faster than other versions of YOLO. To produce predictions, the YOLO model uses a backbone CNN model for feature extraction; here we used the pre-trained ResNet-50 mentioned above. It then decodes the predictions and generates bounding boxes using predefined anchor boxes. For each anchor box, the model predicts a) objectness scores, i.e., the likelihood that a bounding box contains an object, b) anchor box offsets that refine the anchor box position and c) class probability that predicts the class label assigned to each anchor box.

The Faster R-CNN model consists of three main components: the feature extractor, the Region Proposal Network (RPN) and the FC layers. The pre-trained ResNet-50 feature extractor was utilised to generate a fixed-length vector from the input image. Instead of using external algorithms, e.g., edge boxes^52^, to create region proposals, e.g., R-CNN^53^ and Fast R-CNN^54^, the Faster R-CNN utilises the RPN layer to generate bounding boxes using predefined anchor boxes directly within the network. The feature maps were then fed into the region of interest (RoI) pooling layer such that for every proposed region from the RPN layer, the RoI layer maps its corresponding section of the input feature map. Finally, the feature vector from the RoI was fed into classification layers to predict the regression bounding boxes and scores to localise the coordinates of the detected objects and to identify objects (pig postures or drinking), respectively. Table 4 shows the calculated parameters used for pig detection.

**Table 4:** Parameter selection used to train the proposed systems. The exact parameters were used to evaluate each detector proposed in this work; this includes numbers and values of anchor boxes, i.e., pre-defined sets of bounding boxes established to capture the scale and aspect ratio of pigs based on their sizes in each class of the training dataset. All image datasets were resized to match the network input size. The parameters were selected using a nested-cross validation with an independent dataset.

Parameter	Value
Solver	Stochastic gradient descent with momentum optimiser
Momentum	0.9
Learning rate	$1 \times 10^{-3}$
Max number of epoch	5
Size of mini-batch	64
Network input size	[224 224 3]
Number of anchor boxes	[2, 3, 4, 5, 6] boxes values calculated with K-medoids clustering algorithm
Feature extraction network	ResNet-50

#### Anchor boxes

Selecting the number and values of Anchor boxes was proposed to improve the speed and efficiency of detectors^28,29^. During detection, the predefined anchor boxes were tiled across the whole image. Detectors consequently predicted the probabilities and refinements that correspond to each possible pig posture within a tiled anchor box. Here, we introduced predefined anchor boxes^38^, using the K-medoids clustering algorithm with the *IoU* as a distance metric, to enhance system performance in detecting multiple pig postures and pigs with different sizes. The *IoU* between any given two bounding boxes ($Bbox_1$ and $Bbox_2$) is obtained using Eq. (2):

2 $$\begin{aligned} IoU = \frac{Bbox_1 \cap Bbox_2}{Bbox_1 \cup Bbox_2} \end{aligned}$$

Additionally, we used the mean *IoU* of each K-medoids obtained cluster as a quality measure for assessing the estimated anchor boxes; a larger value of *IoU* indicates a better overlap with the boxes in the training data.

#### Pig tracking method

Since our method only detected the behaviour of pigs, multi-pig tracking was performed by utilising these detections at each image frame^55^. We produced a series of time-stamped bounding boxes combined with pig behaviours. These were fed to our designed scheme for pig tracking as follows:

1. We assigned detected pig behaviour to tracks to preserve the identity of pigs across consecutive frame sequence using the Munkres’ variant of the Hungarian assignment algorithm^48^.
2. We initialised new tracks based on unassigned detections. All tracks were initialised as “Tentative”, accounting for the possibility that they resulted from a false detection. This included any emerging pigs to the camera field-of-view.
3. We confirmed tracks only if they had similar vID in a pre-defined number of consecutive frames.
4. We updated existing tracks based on assigned detections.
5. We used Kalman filters to predict existing unassigned tracks^49^.
6. We extracted only valid tracks by removing the un-assigned tracks.

We used the above steps to produce individual pig profiles within pens. In addition to detecting individual pig locations and behaviours, the generated profiles comprised of locomotion activities such as relative speed and distance. The latter metrics were calculated using the Euclidean distance between the centroids, i.e., centre point where the diagonals intersect, of assigned bounding boxes and their relative time (frames).

#### Training and evaluation procedure

In order to build a robust system that generalises to diverse farm settings (e.g., pigs with different colours or sizes) and on pigs exhibiting different intensities of behaviours, we trained the network with varied examples of pigs exhibiting the targeted postures and drinking behaviour. To evaluate our system performance, we used 60% of our dataset for training (68,028 images; 4,41,056 instances) and the rest for testing (45,351 images; 2,94,038 instances). Both training and testing datasets contained images from the routine and the feeding disruption days.

In addition to the large dataset allocated to train the proposed systems, we applied random augmentations to each image batch of the training dataset, i.e., a set of images fed to the detector at a given iteration of the training phase. We repeated this process at each epoch to consistently provide the network with slightly different sets of images. This mechanism prevented our systems from overfitting and learning the exact features at each epoch. The relatively large number of images/instances used for validating the performance of the proposed method, combined with the additional testing dataset which was collected in completely different experimental conditions, enable us to assess thoroughly the generalisation capacities of these models to different scenarios linked with e.g., crowding at specific areas of the pen. To quantitatively evaluate detection performance, we used the mean of the average precision (*mAP*) across all five classes. The average precision for a particular class encompasses both the precision (*p*) and the recall (*r*); it represents the area under the precision-recall curve (Eq. 3) across all test image dataset.

3 $$\begin{aligned} Average\, Precision\, (AP) = \int _{0}^{1}p(r)dr \end{aligned}$$

Additionally, we used the log-average miss rate per each class as another metric to measure the performance of the proposed systems. This metric is similar to average precision (described above); however, it represents pig postures that are not detected.

To evaluate the tracking performance of our system, we used the multi-object tracker accuracy (*MOTA*) metric^56^, described in Eq. (4). This metric involves three sources of error: false negative (*FN*), false positive (*FP*) and identity switch (*IDSW*). False negatives denote pigs that are not tracked, false positives correspond to tracked pigs that do not exist and identity switches represent tracked pigs however with new identities.

4 $$\begin{aligned} MOTA = 1 - \frac{\sum _{t} FN_t + FP_t + IDSW_t}{\sum _{t}GT_t} \end{aligned}$$

where *GT* is the number of ground truth pigs and *t* is the time index of the frame. Multi-Object Tracking Precision (*MOTP*) metric^56^ measures the localisation precision of all true positive detections using the corresponding ground truth annotations as obtained by Eq. (5):

5 $$\begin{aligned} MOTP = \frac{\sum _{i,t}d_t^i}{\sum _{t}c_t} \end{aligned}$$

where *d* is the distance between the target *i* and its corresponding ground-truth annotation measured as the intersection between the bounding boxes, and *c* is the number of targets that correspond to ground truth annotations at time index of frame *t*.

#### Baseline model feature visualisation

To visualise areas of activations of the baseline model that outlines the detection process, we extracted feature maps from a selected layer of our trained ResNet-50 baseline model. Empirical analysis showed that the $37{\mathrm{^{th}}}$ layer of the trained baseline model provides a suitable spatial resolution for visualisation. Finally, we superimposed the entire input image by its resized network map (with maximum activation values) to locate areas of formative features in the original image.

## Supplementary information

Supplementary Information.

## Data Availability

All animal data and code pertaining to this study will be included in the published article. The image dataset including its annotation will also be made available.
