# Supplementary material for: Automated recognition of postures and drinking behaviour for the detection of compromised health in pigs
Source: Sci Rep. 2020 Aug 12;10:13665. doi: 10.1038/s41598-020-70688-6 (PMC7423952; doi:10.1038/s41598-020-70688-6)
Supplement: Supplementary file 1 — Supplementary Information. [file 41598_2020_70688_MOESM1_ESM.pdf]

Automated recognition of postures and drinking behaviour for the detection of compromised health in pigs

Ali Alameer[1,2,*], Ilias Kyriazakis[3,+], and Jaume Bacardit[2,+]

[1] *School of Natural and Environmental Sciences, Newcastle University, Newcastle Upon Tyne, NE1 7RU, UK*
[2] *School of Computing, Newcastle University, Newcastle Upon Tyne, NE4 5TG, UK*
[3] *Institute for Global Food Security, Queen's University, Belfast, BT9 5DL, UK*
[*] *Ali.Alameer@Newcastle.ac.uk*
[+] *these authors contributed equally to this work*

Anchor box size	Anchor box dimension
2	$w = [119; 65], h = [52; 75]$
3	$w = [121; 61; 107], h = [44; 75; 69]$
4	$w = [107; 58; 122; 66], h = [71; 80; 46; 69]$
5	$w = [119; 53; 120; 82; 76], h = [61; 75; 40; 83; 68]$
6	$w = [125; 52; 118; 74; 111; 23], h = [54; 75; 39; 75; 71; 32]$

Supplementary Table S1. Dimensions, *i.e.*, width (w) and height (h), of the obtained sets of anchor boxes. The K-medoids clustering method was utilised to estimate the above values to represent the scale and aspect ratio of pigs across all classes of the training dataset.

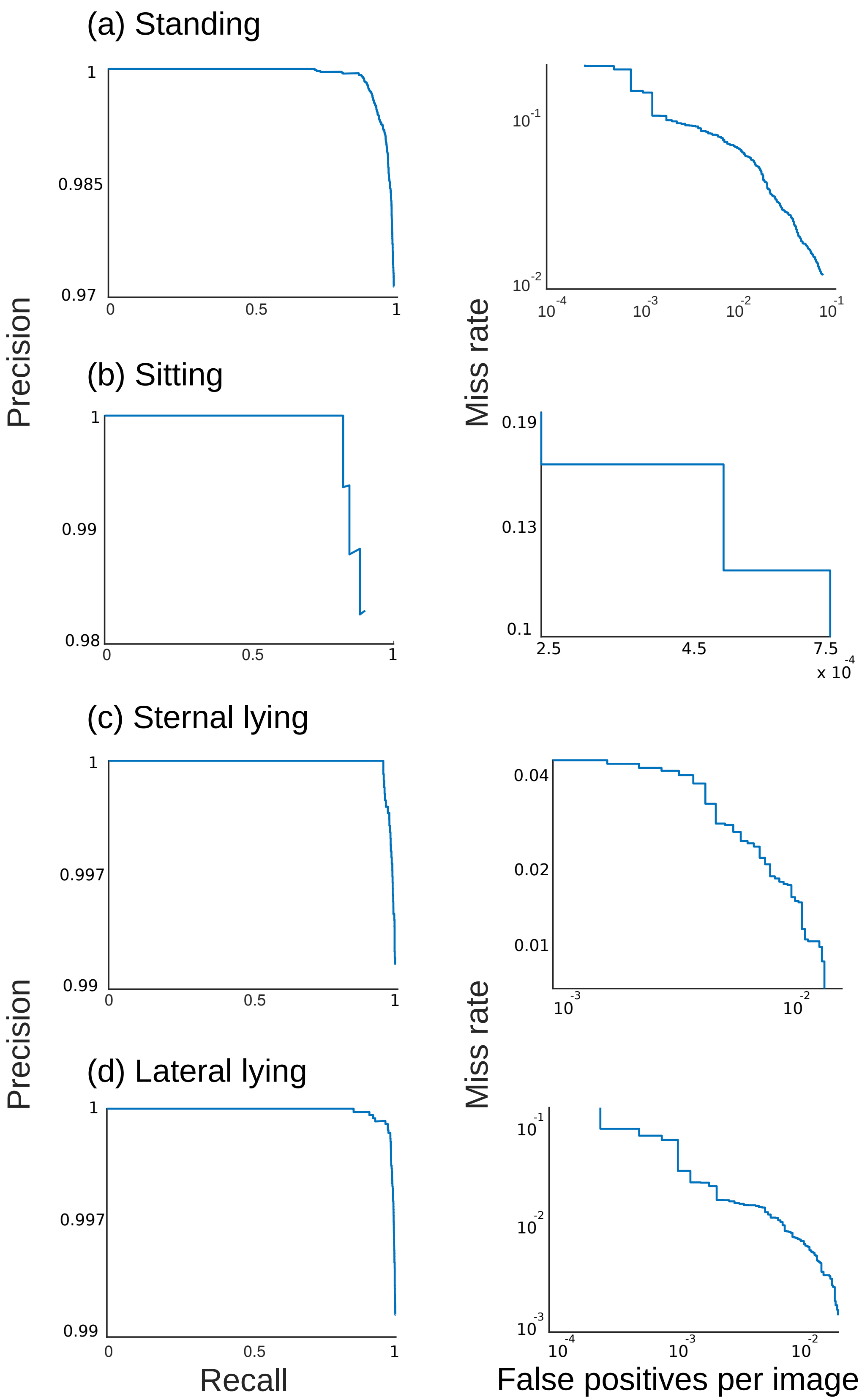

Supplementary Figure S1. The performance of the primary model against data from another commercial pig trial. Left column: precision-recall curve. Right column: miss rate against FPPI; both axes are logarithmically scaled.
